# A comprehensive algorithm for vertical positioning in multi-building environments as an advancement in indoor floor-level detection

**DOI:** 10.1038/s41598-024-64824-9

**Published:** 2024-06-18

**Authors:** Rafał Marjasz, Krzysztof Grochla, Konrad Połys

**Affiliations:** Institute of Theoretical and Applied Informatics, Polish Academy of Sciences, Bałtycka 5, 44-100 Gliwice, Poland

**Keywords:** Floor-level recognition, Indoor vertical positioning, Barometer, GPS, Computational science, Computer science, Information technology

## Abstract

The proliferation of smartphones has catalyzed diverse services, mainly focusing on indoor localization to determine users’ and devices’ positions within buildings. Despite decades of exploration, the seamless integration of wireless technologies in tracking devices and users has become pivotal in various sectors, including health, industry, disaster management, building operations, and surveillance. Extensive research in laboratory and industrial settings, particularly in wireless sensor networks and robotics, has informed indoor localization techniques. This paper, referencing surveys and original literature reviews, proposes an innovative indoor location system amalgamating GPS and barometer readings. GPS identifies entry through doors, while barometer readings facilitate accurate floor-level tracking. The integration promises continuous real-time location updates, enhancing security, navigation, and emergency response. Notably, the algorithm is infrastructure-independent, relying on the smartphone’s barometer, and versatile, detecting elevator travel when Wi-Fi AP or LTE signals are available. Results indicate high accuracy, with building entry exceeding 93%, elevator recognition achieving 75% sensitivity and 97% specificity, and floor change detection surpassing 95% sensitivity and nearly 98% specificity (which translates to nearly 97% accuracy). This comprehensive solution, emphasizing the critical role of precise vertical positioning, signifies an advancement in tracking within urban structures.

## Introduction

The widespread adoption of smartphones and other wireless devices in recent years has given rise to diverse services, with a notable focus on indoor localization. Indoor localization, which involves determining a device’s or user’s location within indoor settings, has been a subject of extensive exploration over the last few decades^1^. However, more than a decade after the widespread proliferation of smartphones and wearable devices featuring wireless communication capabilities, the localization and tracking of these devices have become inseparable from the corresponding tracking of users. This integration has paved the way for various applications and services. Notably, user and device localization holds widespread implications for the health sector, industry, disaster management, building management, surveillance, and various other sectors, emphasizing the critical role of indoor positioning within buildings^2^.

Most of the indoor positioning studies mentioned in survey articles (among others^3–8^) focus on horizontal location within buildings, often altogether omitting or treating the issue of vertical positioning superficially. This trend is apparent, especially in 5 years or older studies; for example, in the article^5^ covering over 140 citations, there is only one publication^9^ strictly relating to floor-level recognition. The cited research works use Angle of Arrival (AoA), Time of Flight (ToF), Return Time of Flight (RTOF) or Received Signal Strength (RSS), which are based on technologies such as Wi-Fi, Radio Frequency Identification of Devices (RFID), Ultra Wideband (UWB), Bluetooth and some other less widespread technologies and systems.

Hence, vertical positioning remains an area that has not been exhaustively researched, especially in the context of vertical location within a multi-building environment. Regarding versatility, a smartphone was chosen as a positioning device based on radio location methods among the available solutions^10–12^. The literature review enabled us to identify areas where a specific gap exists in the research performed and described so far. This fact prompted the authors to conduct research focused exclusively on the following issues: the detection of travels between floors by stairs or the elevator and additionally the detection of entering and exiting the building. Our problem is ensuring continuous monitoring of the occupied floor number while moving between building storeys during distant travels across multiple buildings that are not directly connected. Let us briefly discuss the difficulties and challenges associated with this problem. Firstly, we cannot limit ourselves to the infrastructure installed in buildings, as it may or may not exist in different buildings. Even assuming the broadest possible spectrum of technologies, we may encounter a situation with no infrastructure in a given building. The second difficulty is obtaining information about the building’s topology. In particular, due to the algorithm proposed in the article, it is necessary to obtain knowledge about the height of a single floor in the building and information about which floors the entrance to it is located. Possible challenges regarding buildings with floors with variable heights or mezzanines must also be highlighted here. The last challenge is correcting errors while continuously monitoring the occupied floor number. Due to the continuous functioning of the vertical positioning system, a single error results in a permanent error in determining the position, requiring the most urgent possible correction. Our research introduces a new algorithm designed for precise indoor floor-level detection, filling a research gap in understanding vertical positioning across multiple buildings. Our algorithm stands out by achieving accuracy rates of nearly 94% in identifying floors, surpassing an existing method^13^. Additionally, we enhance functionality by combining various sensing techniques like GPS, barometer readings, and Wi-Fi/LTE signal analysis.

The article is structured into several sections to comprehensively explore the proposed indoor floor-level detection algorithm. It begins with an Introduction, providing an overview of the research topic and objectives. The State of the Art section reviews existing literature on indoor localization, leading to the Conceptualization section of our algorithm. The Background Research elaborates on the algorithm’s draft, followed by Methodology detailing data collection and analysis. Results and conclusions present findings and insights, while Final Remarks discuss implications and future directions. Additionally, Appendix A provides supplementary information on the testbed software used in the study.

## State of the art

Contemporary research works for floor-level recognition based on indoor localization are vastly motivated by the widespread growth of smartphone-embedded sensors. We can distinguish them by the technologies used to determine the floor number. One of the earliest approaches uses the GSM signal-based indoor fingerprinting system^14^. However, the most direct floor-level localization was based on the entity beacon. The proposed system was based on low-cost Radio-frequency identification (RFID) installed on each floor. With a connection to a database, a proprietary location algorithm was implemented in^15^. In^16^, a novel approach to determining the floor of a building is introduced—it uses the pervasive magnetic field measured with the help of a magnetometer present in modern smartphones. The proposed method uses the accelerometer and magnetic data to identify the user’s phone orientation. Afterwards, a Naive Bayes classifier is used. When a specific phone orientation is identified, the magnetic data must be transformed to perform the floor-level identification task. The described approach uses a database of magnetic patterns built in the offline phase of the system.

Among the most popular are the methods that utilize Wi-Fi and barometer. Let us describe a few recent research works. In^17^, a floor-level recognition method utilizes Wi-Fi signals based on the classifier of the support vector machines (SVM). Data for each floor is collected using the smartphone. The fact that the Wi-Fi signal has a noticeable change when passing through walls and floors determines the correct floor number. A similar strategy is used in^18^, where a fingerprint database approach with a Wi-Fi-based floor-level identification method is proposed. The fingerprint grid is constructed, separating each grid point by 1.6 m. Then, based on Fisher’s Linear Discriminant, a floor discriminate model is trained - it selects optimal projection W to maximize the ratio of between-class scatter and within-class scatter. The smartphone accelerometer is used only to identify the user’s state of elevating up or down. A fusion fingerprinting method based on Wi-Fi and barometer was developed in^19^. The so-called BarFi is effective through two main clustering mechanisms: barometer-based hierarchical and Wi-Fi-based K-Means clustering. The design of the BarFi approach uses crowdsourcing as an automatic fingerprint collection method. However, such a fingerprinting approach needs a site survey and back-end server support, limiting its wide application.

The article^20^ concerns the internal and external positioning system on smartphones. The authors propose an indoor-outdoor positioning system using Hidden Markov Models (HMM)-based models to select optimal features for the construction of an environmental perception model. The system uses magnetic field strength as an additional observation feature to reduce latency when switching between different environments. Experimental results show that the perception model can accurately identify environmental changes with a judgment accuracy of over 95%. The article also describes the GNSS/INS/BLE positioning system. The outdoor area uses GNSS positioning algorithms, the indoor area uses BLE positioning, and the transition areas use INS observational data combined with GNSS or BLE for positioning. This system is designed to provide accurate and continuous positioning in a variety of environments. The system has been tested in various environments and configurations, and the results show that the maximum positioning error does not exceed 0.5 m, which proves the high accuracy and reliability of the system. Although the HMM model improves environmental perception, there is a time delay between the recognition of environmental changes. When moving from indoors to outdoors, the smartphone needs time to locate satellite signals, which may delay accurate scenario recognition and lead to a decrease in scenario recognition accuracy. Moreover, in buildings where a lot of reinforced materials are used, internal geomagnetism may be disturbed.

Finally, let us briefly explore the research related closely to the barometer sensor. The paper^21^ discusses the necessary considerations for using barometers for indoor applications based on experiments. A detailed review of the nature of atmospheric pressure is given, concluding with a proposed method for floor positioning. The proposed method consists of Wi-Fi position technology to obtain 2.5D positions (x, y and floor level) and basic information about the indoor environment, such as the difference between floor heights. It is possible to detect the change in floor level with an appropriate threshold.

The authors of the article^22^ point out that although air pressure difference measurements are the basis for floor positioning methods, these methods encounter challenges. First, these methods depend on reference barometers distributed throughout the environment, which limits their scalability. Secondly, air pressure varies, and not just with respect to altitude, which can lead to significant errors in floor positioning, especially since air conditioning systems can significantly affect barometric observations. Finally, there is a serious problem of device heterogeneity; Due to lack of factory calibration, air pressure readings from different smartphone brands may vary, and even readings from two identical devices of the same brand may not be consistent.

In the^13^ article, the authors introduce a detailed method for indoor floor positioning utilizing the smartphone’s barometer. This method translates atmospheric pressure into relative height, enabling the identification of whether a user is ascending or descending stairs and determining their current floor level. Additionally, an entry detection technique is introduced, which establishes whether a user has entered the building, critical for assigning an initial floor location. A key aspect of the method is the calibration of pressure sensors across different devices, which serves to minimize errors resulting from variances in sensor accuracy among different smartphone models. Experimental results, conducted under various conditions and with different devices, demonstrate an accuracy rate exceeding 85% in floor identification, confirming the effectiveness of the proposed solution in real-world application scenarios. This paper adopts a rather general estimation method, which leads to the problem of how to more accurately identify the start and end times of activities to further improve the accuracy of the floor positioning method.

In^23^, a simple appliance, the smartphone’s barometer was used to determine one of two possible user floors. Barometer data were collected at eight different locations on each floor. Next, the average B() of the collected barometer readings was used as a benchmark to determine the user’s floor-level. The corresponding floor is determined if the current barometer reading was within ±0.1 hPa of B(f1) or B(f2). The^24^ show results that while absolute pressure readings have significant time-of-day variations, the difference in pressure across different floor pairs is steady and remarkably consistent for any given building. The authors used the pressure difference as a helpful fingerprint to determine the number of floors changed with close to 100% accuracy. To achieve such accuracy, a 30-sample average (on each floor) was taken to smooth out the noise despite a high measurement device noise. The barometer has proven highly robust to changes in the phone’s on-body placement and orientation, making it an excellent real-life vertical activity detection.

The approach proposed in^25^ was based on a smartphone barometer sensor to identify floor levels in a multistory building. It consisted of two phases: the training phase and the localization phase. The training phase was used to record the smartphone barometer readings for each floor in the building and then sent to the database server for storage. The localization phase was used to determine the floor number the user may occupy. Firstly, the user floor number is determined by extracting the current smartphone barometer reading of the user and then in conjunction with retrieved offline barometer readings during the training phase. In^26^, we find a second system that works on the fingerprint map, built from barometer historical data using a crowdsourcing approach. The barometers are calibrated between all users (for example, during the joint journey in an elevator). The authors conduct a clustering stage of calibrated barometer readings to obtain a fingerprint map of pressures assigned to each floor.

In^27^ work, an approach based on extreme gradient boosting (XGBoost) to recognize five kinds of indoor activities: walking, stillness, stair climbing, escalator, and elevator taking, is taken under research. The authors analyze some specific features in the frequency domain and wavelet domain extracted from the accelerometer, gyroscope, and barometer data to improve recognition accuracy. They do not profoundly investigate the relevant features of the barometer data related to the activities. Article^28^ proposes a floor localization algorithm based on a waist-mounted IMU (inertial measurement unit) and a barometer. First, a climbing stairs activity is detected, and then the vertical displacement is calculated. Following this, a Bayesian network approach is used to infer the floor level change with only rough floor height information. Experiments evaluate the proposed approach and achieve an excellent vertical tracking effect. However, some other stairway structures still lack an apparent landing area and angular variation (i.e., straight, spiral, circular, winding, and abnormity stairs), where the method mentioned above fails.

Article^29^ introduces the application of smartphone sensors. Therein, on each floor, there is a Wi-Fi-based barometer sensor. Reference data from each sensor is retrieved and updated on the AP’s (access points) server. Each time a floor identification is performed, the reference barometric data is compared with the current pressure value on the smartphone. The experiments are performed on three smartphones with ”in-pocket” and ”out-of-pocket” modes. However, in a 5-s window, only just over 94% accuracy can be achieved. This approach requires reference data each time an experiment is performed. It is infrastructure-dependent and must always communicate with the server when obtaining reference barometer pressure values.

The paper^30^ focuses on automatically locating Wi-Fi access points (APs) using data from multiple mobile users. A key aspect is the detection of vertical movements of users, such as climbing stairs or using elevators, which allows the vertical positioning of users’ trajectories and thus more accurate localization of APs. The method uses air pressure measurements to detect changes in floor levels and Wi-Fi received signal strength (RSS) to estimate the location of the AP. Tests were carried out in a real environment using a library building.

The article^31^ focuses on improving indoor altitude estimates using smartphone barometric sensors. The main problem addressed in this study is the effect of ventilation systems on pressure measurements, which may lead to errors in height estimation. The authors propose a correction method that compensates for fan-induced noise by using machine learning to detect changes in the ventilation environment and adjusting height estimates accordingly. The results of experiments conducted in real conditions confirm the effectiveness of the proposed method.

## Conceptualization

The state of the art was analyzed, focusing on floor-level recognition methods, emphasizing the growth of smartphone-embedded sensors. Various technologies, including GSM signal-based indoor fingerprinting, low-cost RFID for direct floor-level localization, and magnetic field measurements using smartphone magnetometers, were explored. Wi-Fi and barometer-based methods are also discussed, highlighting SVM classifiers, fingerprint databases, and clustering mechanisms like BarFi. Noteworthy research utilizes the barometer for floor-level identification, considering atmospheric pressure, pressure differences, and calibration for robust vertical activity detection.

Forming the conception of an indoor vertical location system, we considered the challenges and difficulties mentioned in the introduction. A smartphone was chosen as the device providing information enabling vertical location. We decided that its basic functionality should be completely independent of the infrastructure installed in the building. This fact prompted us to focus our attention on the use of sensors installed in the device, among which the barometer seemed to be the most sensible for detecting changes in altitude. When choosing a method enabling the indication of crossing the building’s entrance threshold, we were also guided by the idea of infrastructural independence, hence our attention to the GPS.

Next, we approached problems related to building topology in the following way. Because most buildings in the world contain floors of regular height, our algorithm, in its basic form, requires measuring and providing this height as a single parameter. In the case of buildings with irregular floor heights, nothing prevents us from considering this fact in an extended version of the algorithm, taking as a parameter the height topology of individual floors. Nevertheless, due to the availability of only standard types of buildings in which the research was carried out, we limited ourselves to the basic form of the algorithm.

The problem of the mezzanines is not the focus of our attention. Firstly, we assume that separating a space forming a mezzanine within a single floor and a given room does not change the number of floors in the building. When positioning the device using horizontal location methods, the expected result is a location within one large room, in which a mezzanine is an integral part of this room. Therefore, the vertical position should also indicate the floor of this large room. A separate issue is a situation in which an existing large room occupies a space two or more floors high, containing a separate mezzanine with a height equal to or greater than a single floor. We then assume that entering the mezzanine is categorized as a change of floor because the change in vertical position is so significant that it seems reasonable to recognize it. Moreover, recording the height of the mezzanine according to a separate height scale, adjusted to the height of the mezzanine and at the same time inconsistent with the heights of the floors in the building, would introduce unnecessary chaos. Therefore, we allow for the possibility that being on such a specific mezzanine may lead to an error in the indicated floor from the point of view of the entire building. Secondly, we assume that we move in the spaces of individual buildings, not in building complexes, which could be connected by connectors, creating spaces between floors at irregular heights that stand out from the topology of the floors.

The conception of an indoor location system integrating GPS and barometer readings is designed to provide accurate vertical positioning information within a building. This concept involves leveraging both technologies’ strengths to enhance storey tracking. Specifically, GPS is utilized to detect entry through a door into a building, while barometer readings are employed to detect movement between floors within the building. To create a practical implementation of this solution, it would require, for example, the development of a mobile application that includes a floor plan/map of the building and information necessary to determine the initial floor level. Additionally, the application could contain so-called ’fingerprint’ data to improve positioning accuracy. We will briefly introduce the problem of localization using the mentioned technologies in the following steps:

- GPS-based entry detection
  - Concept: GPS, or Global Positioning System, is traditionally an outdoor positioning technology. However, the concept here involves using GPS signals near entry points, such as doors or windows, to detect when a person enters a building.
  - Implementation: As a person approaches a building, their smartphone or device equipped with GPS capabilities receives signals from nearby satellites. When the user crosses the threshold (e.g., enters through a door), the drop in GPS signal strength or proximity triggers an entry event.
- Barometer-based floor-level detection
  - Concept: Barometer readings are sensitive to changes in atmospheric pressure, which can be used to infer changes in altitude. In a multi-story building, barometer readings can help determine movement between floors.
  - Implementation: Each floor of a building has a distinct atmospheric pressure level. As a person moves between floors, the barometer sensor in their device detects changes in pressure. Algorithms can then analyze these changes to ascertain vertical movement within the building.
- Integration and location tracking
  - Concept: The system can maintain continuous location tracking by combining GPS and barometer data, providing information on vertical movements within the building and moving between buildings.
  - Implementation: The system integrates the GPS and barometer readings, updating the user’s location in real-time. For instance, it can detect when a person enters the building, determine their floor-level, and track their movement as they navigate different areas within the urban structure.
- Use cases and benefits
  - Enhances security measures by detecting entries and movements between storeys within a building.
  - Facilitates indoor navigation by providing accurate floor-level information.
  - Aids in emergency response scenarios by precise vertical localization of individuals within a building.

The conceptualization introduces a novel indoor location system integrating GPS and barometer readings to provide accurate vertical positioning information. This system leverages GPS for entry detection and barometer readings for movement between floors, offering a comprehensive solution for vertical tracking. The proposed algorithm encompasses three key steps: GPS-based entry detection, barometer-based floor-level detection, and integration for continuous location tracking.

To effectively utilize GPS signal data to determine which building entrance was used, several issues must be addressed. The first step is to create a preliminary map of the building that includes all possible entrances. The map should contain not only the location of the entrances but also other distinctive points that can assist in orientation. The next stage is the so-called fingerprinting process, which involves collecting GPS signal data at various points within the building. Fingerprinting allows for the creation of a database containing unique ”fingerprints” of the GPS signal for different locations, including each of the entrances. In practice, this means recording information about signal strength, available satellites, and other parameters that can vary depending on the specific location. Using this data, an algorithm can then be implemented that compares the current GPS readings of the user with the fingerprint database. This way, even if the accuracy of the GPS is not sufficient to directly indicate a specific entrance (which can occur, especially in urban environments where the signal can be distorted by buildings), the gathered information can be used to estimate which entrance is most likely. Additionally, height data returned by the GPS can be used to further narrow down the possibilities, especially in the case of multi-story buildings. Although the accuracy of GPS height measurements may be limited, it can provide useful hints, especially when combined with other data, such as maps and fingerprinting. Ultimately, this solution requires the integration of various technologies and methods but can offer an effective tool for determining which building entrance was used, based on GPS signal data and a carefully prepared map of the building. This is still only one of the possibilities of using the proposed solution. All described steps can be performed on the target device or in a more comprehensive way, however, our goal is to not require the installation of additional infrastructure.

In summary, an indoor location system based on GPS and barometer readings will offer a comprehensive solution for vertical tracking of individuals inside a building, leveraging GPS for entry detection and barometer readings for movement between floors. This integration enhances indoor positioning accuracy and offers various security, navigation, and emergency response applications.

In a case of application of that solution, the ways of using the algorithm can be very diverse. One of the simplest cases is to download an application that has the appropriate information implemented (e.g. floor plans). Such an application may, for example, assist the user in moving around the building. In this use case, the user does not share data from their device. The application may also have data supporting position detection, the so-called fingerprint, which may also be provided at the stage of its creation or may come from users. In the latter case, data from sensors (e.g. barometer and GPS) could be collected in an anonymized form, but of course with the user’s consent.

## Background research

Notably, the outlined conception for floor-level detection introduces an algorithm that does not rely on fingerprints or building infrastructure. It requires only the information of the approximate height of the floor, making it independent of other specific building characteristics. Using a smartphone’s barometer is sufficient, eliminating the necessity for additional infrastructure like Wi-Fi access points (AP). Additionally, when Wi-Fi AP and LTE are available, the algorithm can detect elevator travel, demonstrating its versatility and potential for practical implementation. This approach departs from traditional methods, offering a promising solution for accurate and infrastructure-independent storey number detection. The draft of the algorithm for indoor floor-level detection involves the following steps:

- (key feature) Utilize the fading of GPS signals near entry points to detect when a person enters a building. (ref.: Methodology: Detection of the presence inside the building)
- (optional feature) Monitor the weakening of Wi-Fi and LTE signals, which often occur while staying in the elevator and during the trip between floors. This can serve as an optional step to distinguish the way of movement between floors. (ref.: Methodology: Elevator ride detection)
- (key feature) Leverage the barometer readings to detect changes in atmospheric pressure, inferring changes in altitude and identifying floor changes. As a person moves between floors, the barometer sensor in their device detects changes in pressure. The algorithm analyzes these changes to ascertain vertical movement within the building and determines the current floor number. (ref.: Methodology: Floor change recognition)
- (optional feature) Periodically check the consistency of the current floor number with the actual. This involves either the presence of access points (or other location markers) on that floor or requires human intervention in manually correcting the floor number.

In summary, the proposed algorithm ensures tracking of vertical movements within a building by combining GPS, barometer readings, and optional Wi-Fi/LTE signal analysis. The optional elevator trip detection adds flexibility to the algorithm, providing a more comprehensive solution for indoor floor-level recognition.

The measurements were collected to determine whether the detection of entry, elevator ride, or change of floor number occurred. The rationale for conducting measurements in our research stems from the necessity to address the challenges associated with indoor localization, particularly regarding vertical positioning within buildings. The utilization of the outlined algorithm draft is motivated by the need for accurate and infrastructure-independent floor-level detection without relying on fingerprints or building infrastructure in a multi-building environment.

**Figure 1:**
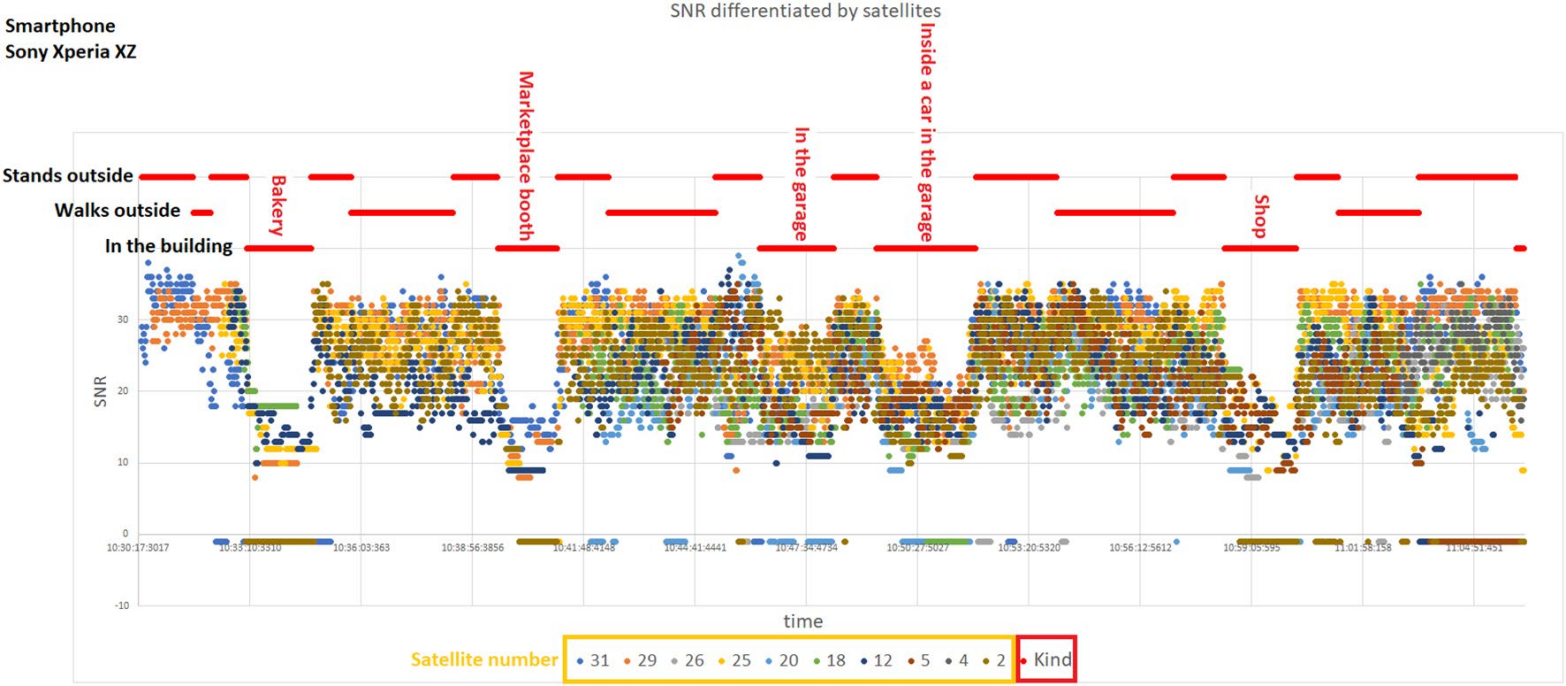
Example of measurements taken at the housing estate located in Ruda Śla̧ska city.

**Figure 2:**
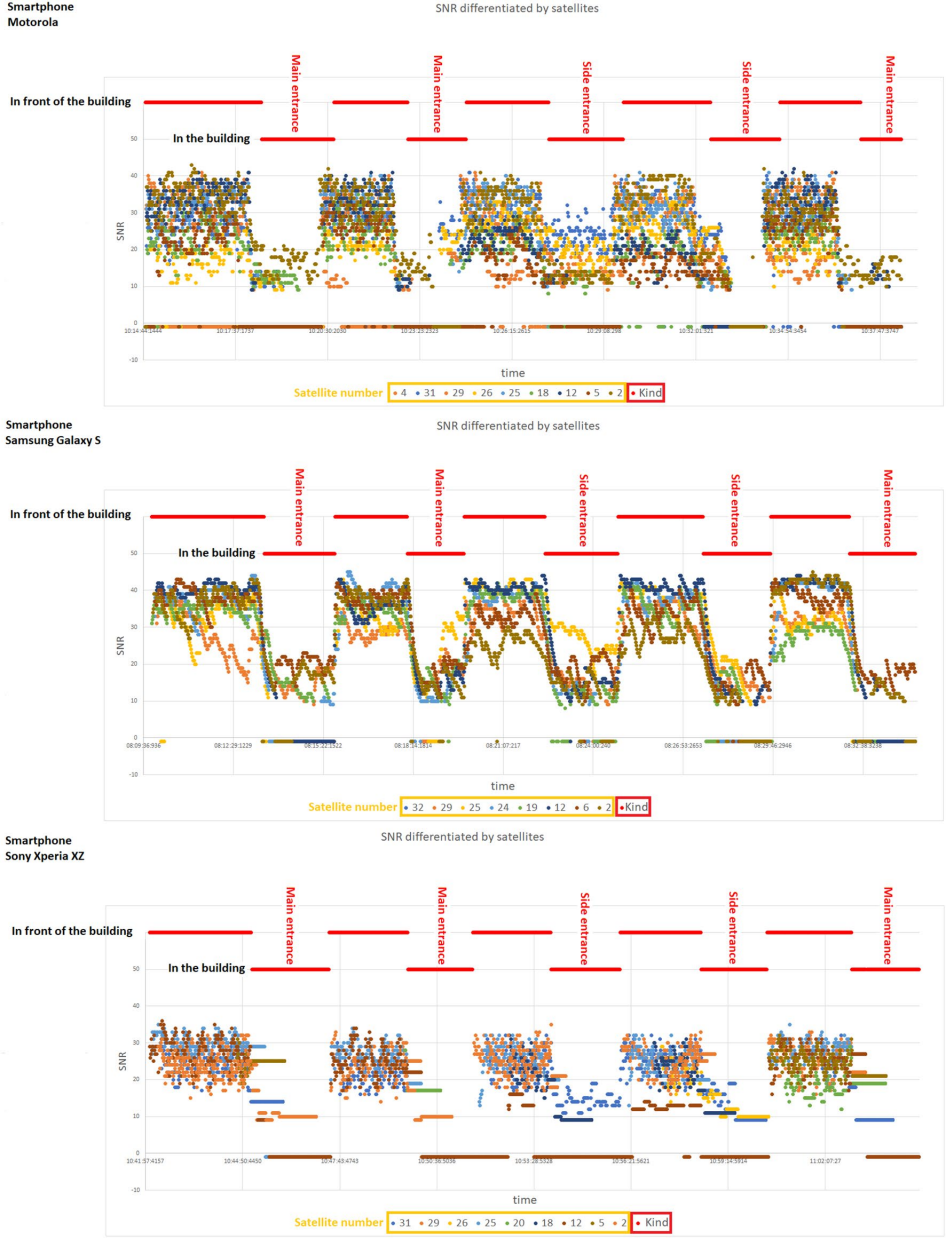
Example of GPS measurements taken at the PAS Institute.

**Figure 3:**
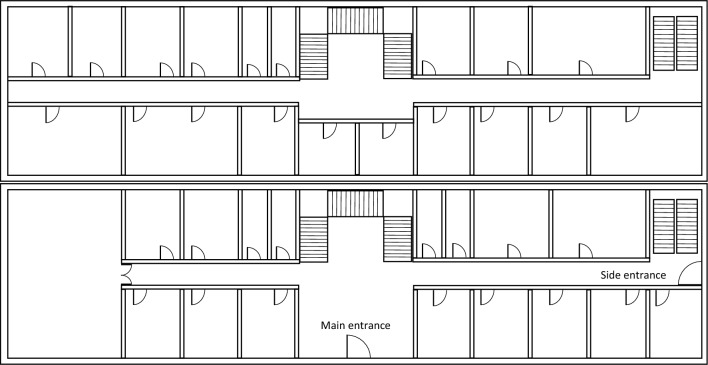
Scheme of ground and higher floors of the PAS Institute building.

The following scenarios were used to detect entering and exiting the building. The measurements were collected using four smartphone models: Huawei P30 Pro Android 10, Motorola E6s Android 9, Samsung Galaxy S7 Android 8, and Sony Xperia XZ Android 8:

- entering and exiting the building of the Polish Academy of Sciences Institute (PAS Institute) through the main entrance and the side entrance (floor plan is depicted on Fig. 3),
- walking on a housing estate located in the city of Ruda Śla̧ska, including visiting local buildings.

The measurements include:

- height of recorded GPS satellites,
- azimuth of recorded GPS satellites,
- SNR level of the signal of recorded GPS satellites.

Measurements from the GPS module were recorded with a frequency of 1 Hz. In Figs. 1 and 2 we present a samples of collected data.

The following scenarios were used to detect the elevator travel. The measurements were collected using a Samsung Galaxy S7 Android 8 smartphone:

- riding the elevator combined with leaving the elevator on each floor,
- walking on the stairs with a stop on each floor.

The measurements include:

- pressure indications from the barometer,
- LTE received signal strength indicator,
- Wi-Fi received signal strength indicator.

**Figure 4:**
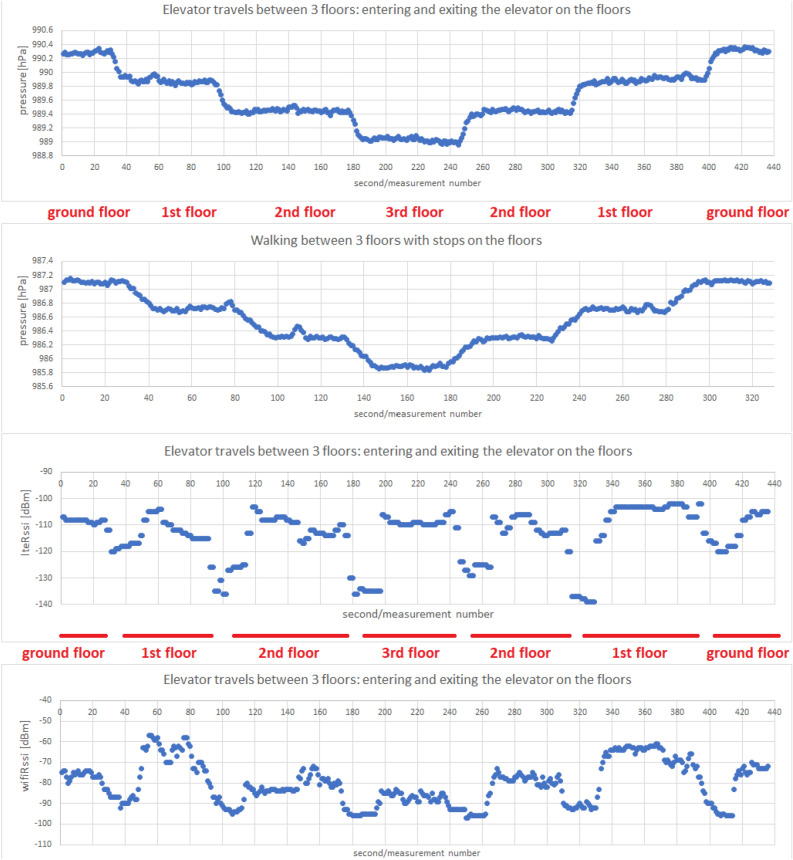
Example of barometer pressure, LTE RSSI and Wi-Fi RSSI measurements taken at the PAS Institute.

Measurements were recorded with a frequency of 1 Hz. In Fig. 4 we present a sample of collected data. We observe a clear trend of changes in the value of the recorded pressure depending on the movement between floors. For an elevator traveling vertically at a pace that differs from climbing stairs, the pressure drop with height is slightly more pronounced than the gentler drop observed in the staircase. The collected measurements are divided by four days of difference (the walking measurement was made on March 19, the elevator ride measurement on March 23)—this fact affects the differences in the pressure values observed.

The following scenarios were used, according to which the measurements were collected using two smartphone models—Samsung Galaxy S7 Android 8 and Sony Xperia XZ Android 8:

- lift travel combined with leaving the elevator on the chosen floors in the PAS Institute,
- walking up and downstairs with long or short stops on each floor: in an apartment building located in the city of Ruda Śla̧ska and in the PAS Institute,
- picking up the phone from the table to your hand, holding it in your hand for several seconds, and putting it back on the table,
- a telephone placed on a table or in hand, windows on opposite sides of the room are opened then closed.

**Figure 5:**
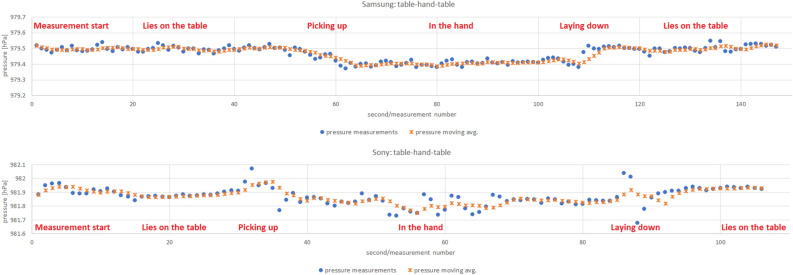
Example of barometer pressure measurements taken according to a scenario of picking up and putting the phone on the table.

**Figure 6:**
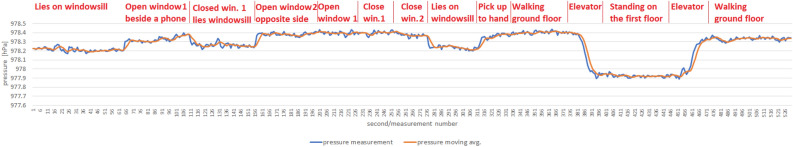
Example of barometer pressure measurements taken according to a scenario considering window opening and closing.

The measurements include pressure indications from the smartphones barometer. Measurements were recorded with the same frequency of 1 Hz. In Figs. 5 and 6 we present a samples of collected data.

## Methodology

### Detection of the presence inside the building

In the case of detection of entering and leaving the building, the literature describing the issue under study describes the significance of the satellite altitude parameter. The article^32^ documents the impact of the satellite height on the quality of the signal strength drop recorded inside the building. This fact suggests the need to distinguish between the satellites according to their height and use a method that considers the different characteristics of the decrease in signal strength from individual satellites. However, in the measurements collected during the research, about ten satellites in the range were recorded, of which the SNR measurements in a given second came on average from seven satellites (in the worst moments, they came from only four satellites). Taking into account such a small number of GPS signal sources, we abandoned the idea of complete rejection of the signal of low-altitude satellites in favor of a solution sorting the satellites with the SNR level (this approach takes into account the height of the satellite in relation to the receiver through a more minor or more significant decrease in the SNR value depending on this height). The sorting is done by calculating the $\text {SNR}_{p_n}$, which is the n-th percentile of the recorded SNR in each second. The n-th percentile characteristics depend on the signal recorded inside and outside the buildings. They are representative of the total number of satellites within the range of the smartphone because the example of a temporary loss of signal to one satellite does not affect the inability to calculate the percentile and is compensated by the remaining undisturbed at that moment satellites.

**Figure 7:**
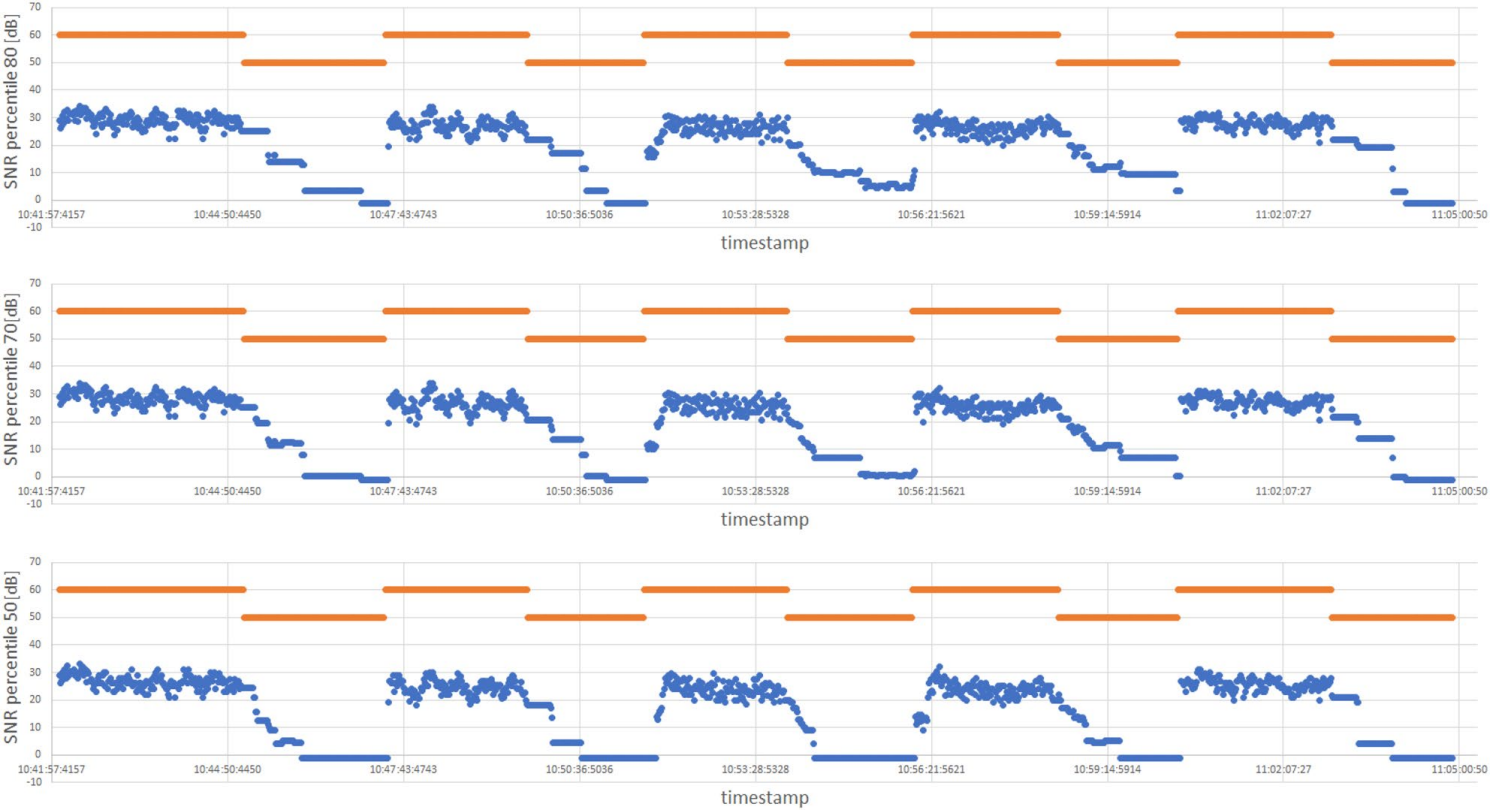
Visualization of the characteristics of example $\{\text {SNR}_{p_{50}},\text {SNR}_{p_{70}},\text {SNR}_{p_{80}}\}$ percentile values. Blue dots represent SNR percentile values, while the orange lines indicate the periods when the smartphone was inside (lower lines) and outside (higher lines) the building.

Figure 7 presents graphs of exemplary $\text {SNR}_{p_n}$ values in relation to the state of being inside or outside the PAS Institute’s building. We observed a significant correlation and analyzed it by calculating the sensitivity and specificity of the diagnostic test to determine the state of being in a building by comparing the SNR percentile value to the adopted $\text {SNR}_{T}$ threshold. Sensitivity and specificity are commonly used metrics^33,34^ in binary classification tasks, such as identifying positive and negative cases in medical diagnoses or detecting events in sensor data. Sensitivity, also known as the true positive rate, measures the proportion of actual positive cases correctly identified by a model or algorithm. It indicates how well the model identifies true positives and is calculated as the number of true positives divided by the sum of true positives and false negatives. Specificity, on the other hand, measures the proportion of actual negative cases correctly identified by a model. It indicates how well the model identifies true negatives and is calculated as the number of true negatives divided by the sum of true negatives and false positives. Confidence intervals (CI) for 95% confidence were counted for the results obtained using the Wilson method^35^.

**Algorithm 1:**
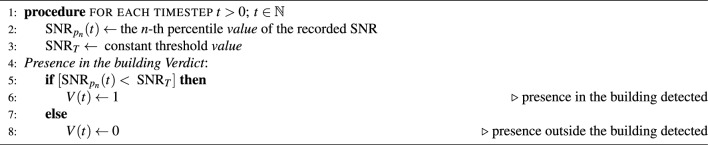
Detection of the presence inside the building

To summarize, our Algorithm 1 of detecting entry into the building is based on the following condition: whenever $[\text {SNR}_{p_n}(t) < \text { SNR}_{T}]$, we categorize the situation as presence inside the building. Otherwise, we classify the situation as being outside of the building.

#### Elevator ride detection

**Figure 8:**
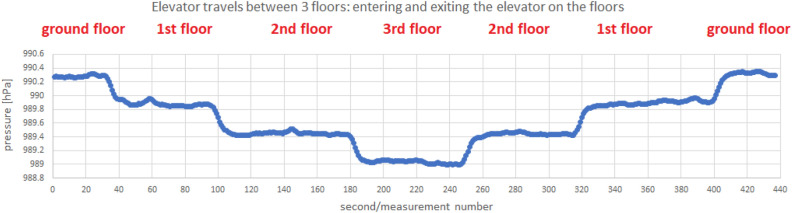
Example of moving average ${\overline{P}}_N$ calculated on barometer pressure, measurements taken at the PAS Institute.

**Figure 9:**
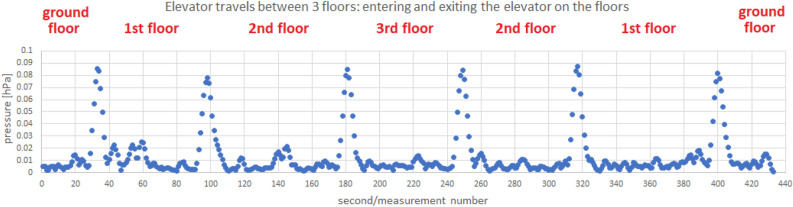
Example of the standard deviations $\sigma _N({\overline{P}}_N)$ calculated from the most recent *N* values of moving average on barometer pressure, measurements taken at the PAS Institute.

Let us progress to the issues connected to the pressure measurements. The observed fluctuations in the value of the recorded pressure during standing on the floors make it hard to draw proper conclusions based on single measurements. We use the moving average ${\overline{P}}_N$ of the most recent *N* measurements to obtain greater pressure consistency - thus, *N*’s value is the first parameter of the constructed method. An example of a moving average value from the most recent five measurements is presented in the Fig. 8. Next, the standard deviations $\sigma _N({\overline{P}}_N)$ from the most recent *N* values were calculated for pre-prepared averaged pressure values (i.e. the standard deviation was calculated from the previously calculated moving averages), resulting in the dependence visible in the following Fig. 9.

**Algorithm 2:**
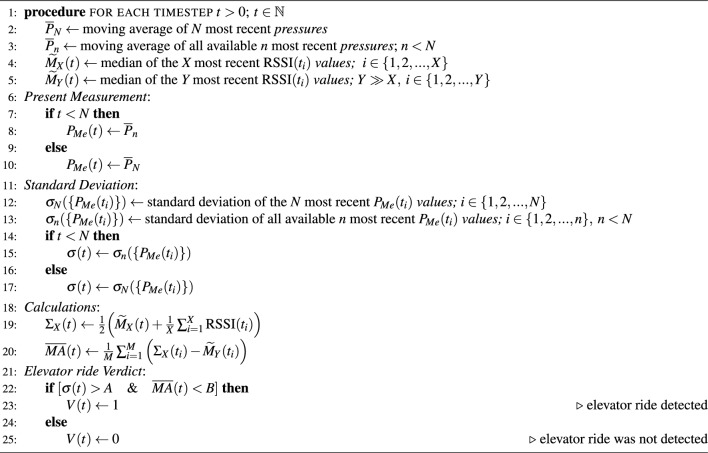
Elevator ride detection

Increased values of the standard deviation indicate the movement moments between floors. The problem remains to determine the deviation limit above which we classify the journey. Let us define this limit by the parameter *A*, the finding of which will be the subject of the analysis of the values of sensitivity and specificity achieved by the created method.

Let us advance to the issue of elevator travel detection. LTE and Wi-Fi signal strengths presented in the Fig. 4 in RSSI values show the dependence of the recorded values decrease on being inside a metal elevator (creating a specific Faraday cage) and irregular oscillations outside the elevator. As in the case of the pressure value, a method of follow-up detection of changes in RSSI values suggesting staying in an elevator was developed (it is independent of the scale of values taken by RSSI, which differs by several dozen decibels for Wi-Fi and LTE signals). For this, we calculate two quantities:

- divided by two the sum of the moving average and the median ${\widetilde{M}}_X$ of the most recent *X* values of RSSI—we denote it with $\Sigma _X= \frac{1}{2}\Big ({\widetilde{M}}_X+\frac{1}{X}\sum _{i=1}^{X}\text {RSSI}(t_i)\Big )$,
- the median of the *Y* most recent RSSI values (our intention is for *Y* to be several times greater than *X*, we motivate it by the desire to juxtapose the short-term and long-term measures)—we denote it with ${\widetilde{M}}_Y$.

**Figure 10:**
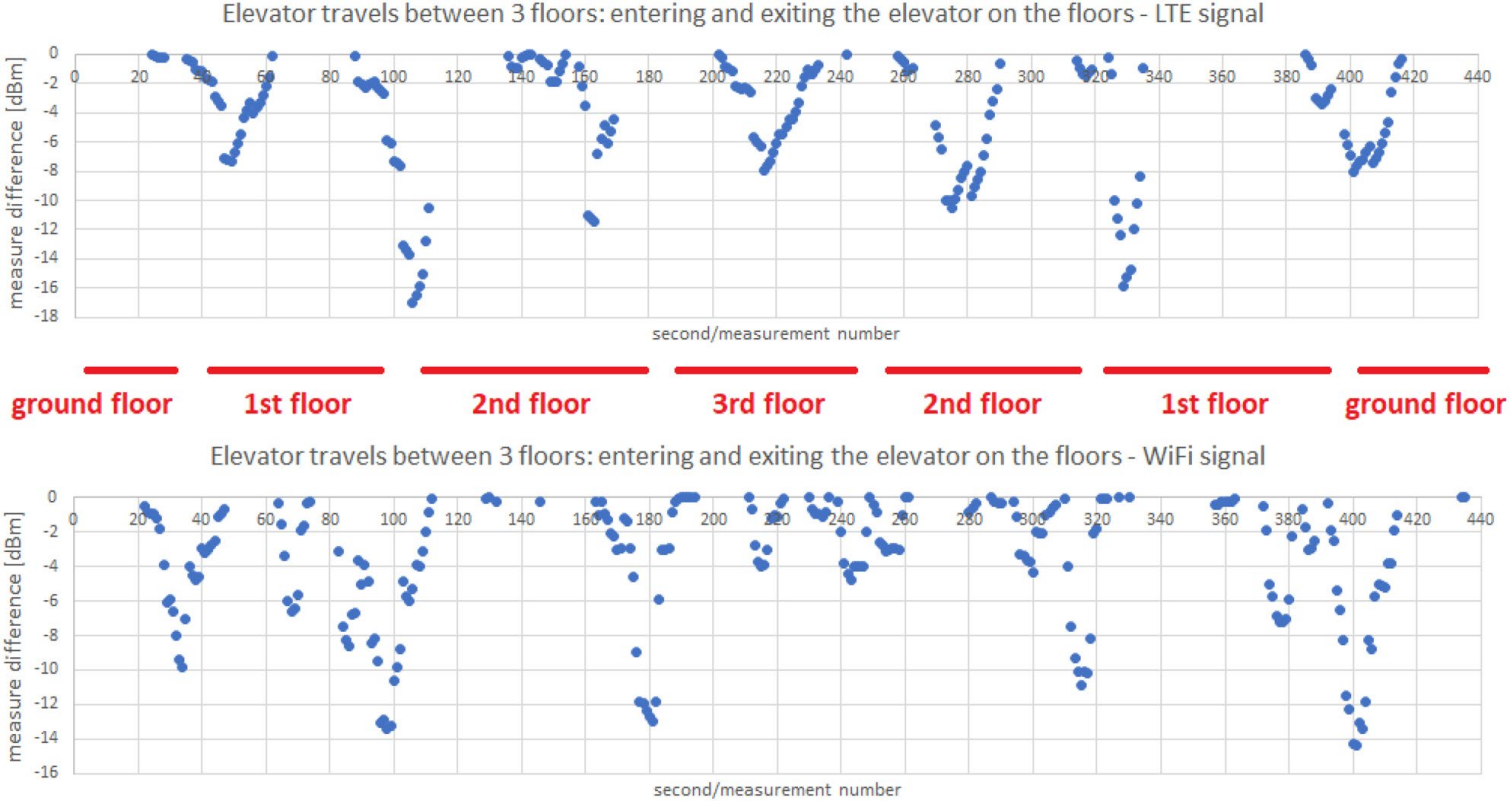
Example of the $\Sigma _X-{\widetilde{M}}_Y$ difference values calculated from LTE and Wi-Fi measurements taken at the PAS Institute.

Then, the difference $\Sigma _X-{\widetilde{M}}_Y$ between both values is examined, the value of which is the lowest during the stay in the elevator. In Fig. 10, we present an example of a graph of values of the discussed difference (independently for RSSI of Wi-Fi and LTE signals).

A large oscillation in value characterizes the proposed measure of difference. We use the moving average of the *M* last differences to prevent this from happening, thus adding another variable to the parameter list. As in the case of the pressure-related method, the problem remains to establish the moving average limit with *M* most recent differences, below which we classify the stay in the elevator. Let us define this limit by parameter *B*, the finding of which will be the subject of our further analysis. To summarize, our elevator ride recognition method can be written in the form of the Algorithm 2.

#### Floor change recognition

**Figure 11:**
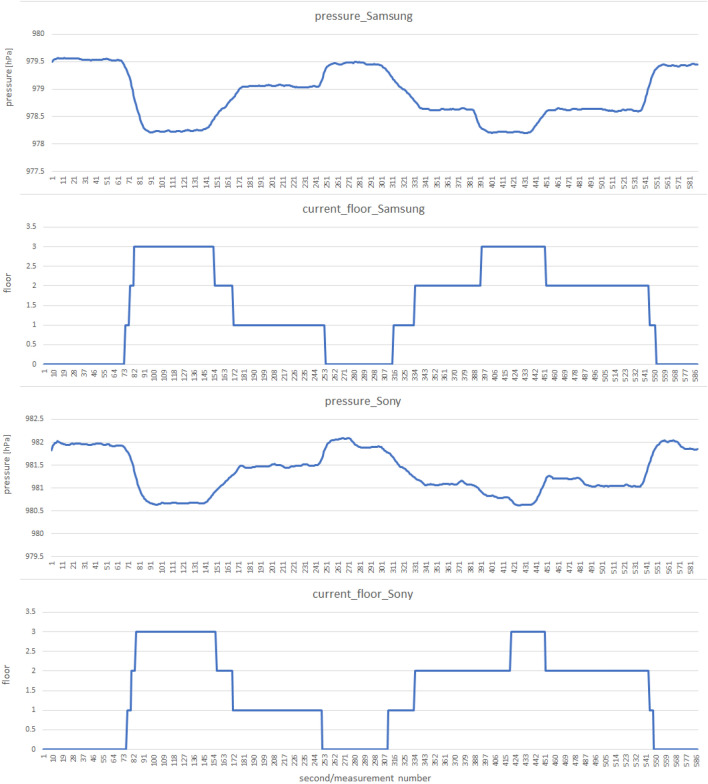
Example of storey number recognition outcome (short period of nearly 10 min).

**Figure 12:**
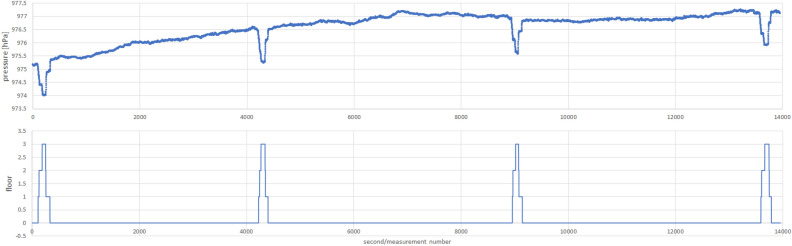
Example of storey number recognition outcome (long period of over 3 h and 53 min).

Let’s finish with issues related to detecting changes in floor numbers indicated with the help of a barometer. At first, we considered the cases where there is a possibility of change in pressure inside the building not related to the movement between the floors. Tests of picking up and putting the phone on the table are visualized in Fig. 5. The amplitudes of pressure changes are moderate (up to 0.2 hPa), which gives a chance for their proper filtering/distinguishing from measurements indicating a change of altitude connected to a building floor (presented on 4). Next, we conducted tests considering window opening and closing compared to the elevator ride taken right after. In Fig. 6, we observe pressure changes caused by the performance of subsequent activities. The amplitude of these pressure changes (again around 0.2 hPa) is distinguishable from the changes (around 0.4 hPa as seen in Figs. 11 and 12) caused by the floor change through elevator rides in the building.

**Algorithm 3:**
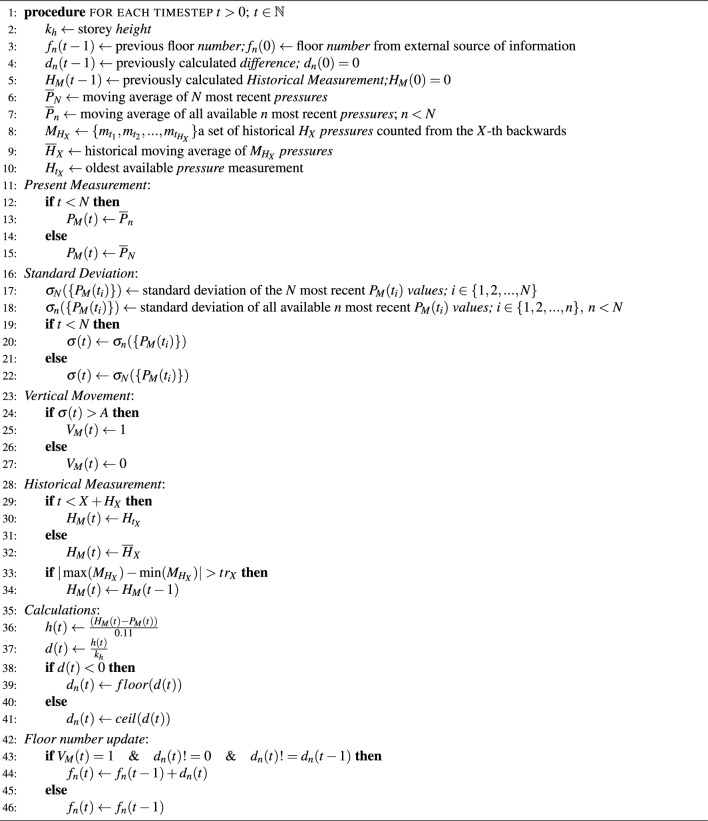
Floor change recognition

As in the case of the presence inside the elevator detection method, the constructed floor change detection method will consist of several steps taken in a set order:

- We calculate the moving average of the most recent *N* measurements. Let’s call it the Present Measurement ($P_M$).
- The standard deviation of the most recent *N* pre-prepared in the previous step averaged pressure measurements is calculated. Based on its value, we deliver our verdict $V_M$ on vertical movement. If the deviation is higher than threshold *A* we classify it as $V_M=1$ (vertical movement occurs), otherwise $V_M=0$ (no vertical movement detected).
- The historical moving average ${\overline{H}}_X$ is calculated from the $H_X$ measurements counted from the *X*-th backwards (e.g. $H_X = 10$, $X = 40$ means that at time $t = 0$ we would calculate the average of ten measurements $\{m_{t_1}, m_{t_2},..., m_{t_{10}}\}$ recorded at times $t_i \in \{-40, -41, -42,..., -49\}$). When it cannot be counted yet (as in the presented case because of too short time from the start of taking the measurements at $t = 0$), we take the oldest known measurement as the first historical mean value (it is the measurement recorded at $t = 0$). Let’s call this value the Historical Measurement ($H_M$).
- We enter the threshold value $tr_X$. Suppose the absolute difference between the maximum and minimum from the measurements $M_{H_X}=\{m_{t_1}, m_{t_2},..., m_{t_{H_X}}\}$ used for calculating the moving average ${\overline{H}}_X$ is greater than $tr_X$. In that case, we consider the historical measurements to be burdened with an error resulting from the ongoing change of storey and use the previous $H_M$ value in further steps.
- As mentioned in article^21^, the values of pressure at various heights above sea level, according to the International Civil Aviation Organization ‘Standard Atmosphere’ model, drop by 0.11hPa for every meter increase in height. We use this value to calculate height resulting from the difference between $H_M$ and $P_M$ values following the formula $h=\frac{(H_M-P_M)[\textrm{hPa}]}{0.11 [\textrm{hPa}/\textrm{m}]}$.
- In the next step, we divide the obtained height by the measured storey height $k_h$ following the formula $d=\frac{h}{k_h}$. If *d* is negative we calculate the difference of storeys as $d_n=floor(d)$ and if positive $d_n=ceil(d)$ is computed.
- By compiling steps 2. and 6., if $V_M=1$ and $d_n!=0$ we detect a change in the occupied floor.
- Finally, we memorize two values: current floor number $f_n$ and floors climbed number $d_n$. Each time $d_n$ changes its value we update $f_n$ accordingly to the occurred change, memorizing new $f_n$ and $d_n$ afterwards.

To summarize, our floor change detection method can be written in the form of the Algorithm 3.

## Results and discussions

### Detection of the presence inside the building

The developed method in the form of Algorithm 1 allows us to determine the presence inside the building with high accuracy, exceeding 93% and reaching almost 99% for some smartphone models. As part of the search for the values of the $\text {SNR}_{T}$ parameter and the SNR percentile, for which the highest values of sensitivity and specificity are simultaneously achieved, all possible combinations of parameters from the following ranges were calculated:

- discrete range of $\text {SNR}_{p_n}$ percentiles for $n\in \{50 \text {-} 100\}$ with step 5,
- discrete $\text {SNR}_{T}$ threshold interval {5-35} with step 1.

The best results obtained are summarized in Table 1. Due to the measurements of the stroll within the residential area, it was found that the pair of parameters 70 percentile $\text {SNR}_{p_{70}}$ and 23 for the $\text {SNR}_{T}$ threshold are the most representative for the whole. The sensitivity and specificity calculations in determining the presence in the building for this set show that the loss between the best results for a given phone amounts to a maximum of 4%.

**Table 1:** Sensitivity and specificity results calculated for recognition of the ”inside the building” state.

$\text {SNR}_{p_n}$	$\text {SNR}_{T}$	Sensitivity result	CI sensitivity	Specificity result	CI Specificity	Smartphone and place
70	23	95.4%	[95.31%, 95.48%]	93.9%	[93.82%, 94.00%]	Sony, estate
70	23	97.7%	[97.56%, 97.84%]	88.8%	[88.65%, 88.90%]	Sony, Institute
70	23	99.7%	[99.55%, 99.83%]	98.1%	[97.92%, 98.20%]	Motorola, Institute
70	23	93.4%	[93.29%, 93.54%]	99.6%	[99.50%, 99.77%]	Samsung, Institute
70	23	99.6%	[99.24%, 99.99%]	98.8%	[98.45%, 99.20%]	Huawei, Institute
The best result for each smartphone individually						
70	23	95.4%	[95.31%, 95.48%]	93.9%	[93.82%, 94.00%]	Sony, estate
80	22	94.3%	[94.12%, 94.39%]	94.3%	[94.18%, 94.45%]	Sony, Institute
80	24	99.2%	[99.08%, 99.36%]	99.1%	[98.96%, 99.23%]	Motorola, Institute
70	33	97.6%	[97.52%, 97.78%]	97.5%	[97.33%, 97.59%]	Samsung, Institute
70	24	100%	[99.62%, 100.00%]	98.8%	[98.45%, 99.20%]	Huawei, Institute

#### Elevator ride detection

The proposed Algorithm 2 of elevator ride recognition gives promising results. Due to the time needed to travel from one floor to the next, determined during the research, oscillating around 30 s, the limit values were 5 and 35 measurements, respectively, for the ”short” and ”long” range of calculating the moving averages and the median. Let’s list all the defined parameters and appropriate ranges of values for them:

- $N \in \{1,\ldots ,5\}$ with step 1—the number of pressure measurements from which we calculate the moving average;
- $A \in \{0.01,\ldots ,0.02\}$ with step 0.001—limit of the standard deviation $\sigma _N({\overline{P}}_N)$ of the most recent N moving average values of the N pressure measurements;
- $X \in \{1,\ldots ,5\}$ with step 1—the number of RSSI measurements of LTE or Wi-Fi signals, from which we calculate $\Sigma _X$;
- $Y \in \{15,\ldots ,35\}$ with step 1—the number of RSSI measurements of LTE or Wi-Fi signals from which we calculate ${\widetilde{M}}_Y$;
- $M \in \{1,\ldots ,5\}$ with step 1—the number of $\Sigma _X-{\widetilde{M}}_Y$ differences from which we calculate the moving average;
- $B \in \{-3.0,\ldots ,0.0\}$ with step 0.05—the moving average limit for the *M* most recent $\Sigma _X-{\widetilde{M}}_Y$ differences.

The analysis results take into account the care for the simultaneous maximization of the percentages achieved by the sensitivity and specificity. We used all possible combinations of parameters from the listed ranges. Table 2 presents the parameter sets for which the two best results are achieved.

The obtained sensitivity value at the level of 75% is moderately good, which, combined with a specificity of about 97%, gives certainty of the verdict regarding the presence in the elevator. Studies with glass elevators have not been conducted—our research concerns only metal elevators, narrowing the method’s applicability. Nevertheless, our further research will concentrate on the best possible storey recognition regardless of the method of moving between floors.

**Table 2:** Sensitivity and specificity results calculated for recognition of the ”inside the elevator” state.

*N*	*A*	*X* LTE	*Y* LTE	*M* LTE	*B* LTE	*X* Wi-Fi	*Y* Wi-Fi	*M* Wi-Fi	*B* Wi-Fi	Sensitivity	CI Sensitivity	Specificity	CI Specificity
5	0.011	5	20	5	-2.2	5	25	5	-2.2	75.3%	[75.09%, 75.50%]	96.8%	[96.56%, 97.11%]
5	0.01	5	27	5	-2.7	5	27	5	-2.7	74.1%	[73.92%, 74.32%]	97.2%	[96.90%, 97.45%]

#### Floor change recognition

Ultimately, for the issue related to detecting a change in floor number, a list of all defined parameters and appropriate ranges of values, among which we will look for a combination providing the best sensitivity and specificity of the proposed Algorithm 3, presents as follows:

- $N \in \{3,\ldots ,10\}$ with step 1—the number of pressure measurements from which we calculate the moving average (the parameter role is the same as in the Algorithm 2);
- $A \in \{0.01,\ldots ,0.02\}$ with step 0.001—limit of the standard deviation of the most recent N moving average values of the N pressure measurements (the parameter role is the same as in the Algorithm 2);
- $X \in \{15,\ldots ,45\}$ with step 5—pressure measurements setback in time value;
- $H_X \in \{5,\ldots ,15\}$ with step 1—the number of pressure measurements from which we calculate the $H_M$ moving average;
- $tr_X \in \{0.05,\ldots ,0.5\}$ with step 0.05—threshold value for the absolute difference between the maximum and minimum from the $H_X$ measurement values.

We used all possible combinations of parameters from the listed ranges. The analysis results take into account the care for the simultaneous maximization of the percentages achieved by the sensitivity and specificity. Table 3 presents the parameter sets for which the two best results are achieved.

**Table 3:** Sensitivity and specificity results calculated for recognition of the ”change in the occupied floor” state.

*N*	*A*	*X*	$H_X$	$tr_X$	Sensitivity (%)	CI Sensitivity	Specificity	CI Specificity
4	0.01	15	13	0.15	95.3	[94.84%, 95.86%]	97.8%	[97.30%, 98.35%]
5	0.01	15	15	0.15	95.4	[94.85%, 95.87%]	97.5%	[96.96%, 98.01%]

The developed Algorithm 3 allows us to determine the change of the occupied floor number inside the building with high accuracy, exceeding the level of 95% for sensitivity value and reaching a specificity of almost 98%. Moving between floors includes both elevator travel and stairs. An example visualization of the method’s application in the PAS Institute’s building is shown in Figs. 11 and 12.

The general applicability of the proposed floor change recognition method is closely linked to the quality of sensors integrated into smartphones, as evidenced by our results. For instance, the Samsung Galaxy S7 exhibited remarkably high accuracy levels, with sensitivity reaching 100% and specificity of almost 99%. Conversely, the Sony Xperia XZ displayed lower accuracy, achieving a sensitivity exceeding 91% and specificity approaching 95%. These findings underscore the significant impact of smartphone sensor quality on the algorithm’s performance, highlighting the importance of considering device variability and sensor characteristics in real-world implementation scenarios.

As we can see in the short trace example, there is a noticeable difference between smartphones in the moments when a floor change is detected. This impact is reflected in the measures of sensitivity and specificity (those few per cent missing from perfection), but it does not affect the detection of a floor number change. The long trace example illustrates atmospheric pressure fluctuations over less than 4 h. These fluctuations are so slow that they do not affect the determination of the storey number (due to the moving averages used in the method). There is a particular limitation of the method—if there is a long enough break in the current pressure measurement, its sudden appearance may cause a sudden jump in the floor number due to the last historical pressure measurement remembered, which may differ significantly from the current one.

An essential component contributing to the method’s robustness is the optional mechanism for periodic verification of the occupied floor number. This element serves as a critical feature in correcting sporadically occurring errors that may emerge during the tracking process. Periodically cross-referencing the estimated floor number with the actual environment, utilizing manual human intervention, Wi-Fi access points or other location markers, ensures the algorithm’s ongoing precision. By incorporating this optional verification mechanism, the method goes beyond merely detecting and correcting immediate changes in floor levels. It establishes a continuous feedback loop, enhancing the algorithm’s resilience against transient discrepancies that may arise due to environmental factors, signal interference, or other unpredictable conditions.

## Summary and future work

Our conception of an algorithm for indoor floor-level detection presents a comprehensive system for precise vertical positioning within buildings. Our study addresses a research gap in the context of vertical positioning within a multi-building environment. While indoor positioning has been extensively studied, vertical positioning remains relatively underexplored, especially across multiple buildings. Our research aimed to fill this gap by proposing a comprehensive algorithm for indoor floor-level detection, explicitly targeting the challenges associated with continuous monitoring of the occupied floor number during travels between buildings and their storeys. By leveraging smartphone-based positioning methods and considering scenarios where infrastructure and building topology information may be limited, our study contributes novel insights and solutions to enhance indoor positioning accuracy and reliability in complex urban environments.

Focusing on finding published solutions for an indoor vertical positioning method based on a smartphone barometer, we have come across one very closely related solution. Our algorithm for indoor floor-level detection, as demonstrated by the sensitivity and specificity results achieved, outperforms the existing method’s accuracy of 85% discussed in^13^. With sensitivity value exceeding 91% and specificity reaching nearly 95% for a smartphone model with a lower quality sensor, our algorithm showcases superior accuracy just under 94% in floor identification tasks. Furthermore, our approach offers enhanced precision by combining multiple sensing modalities, including GPS, barometer readings, and optional Wi-Fi/LTE signal analysis.

**Figure 13:**
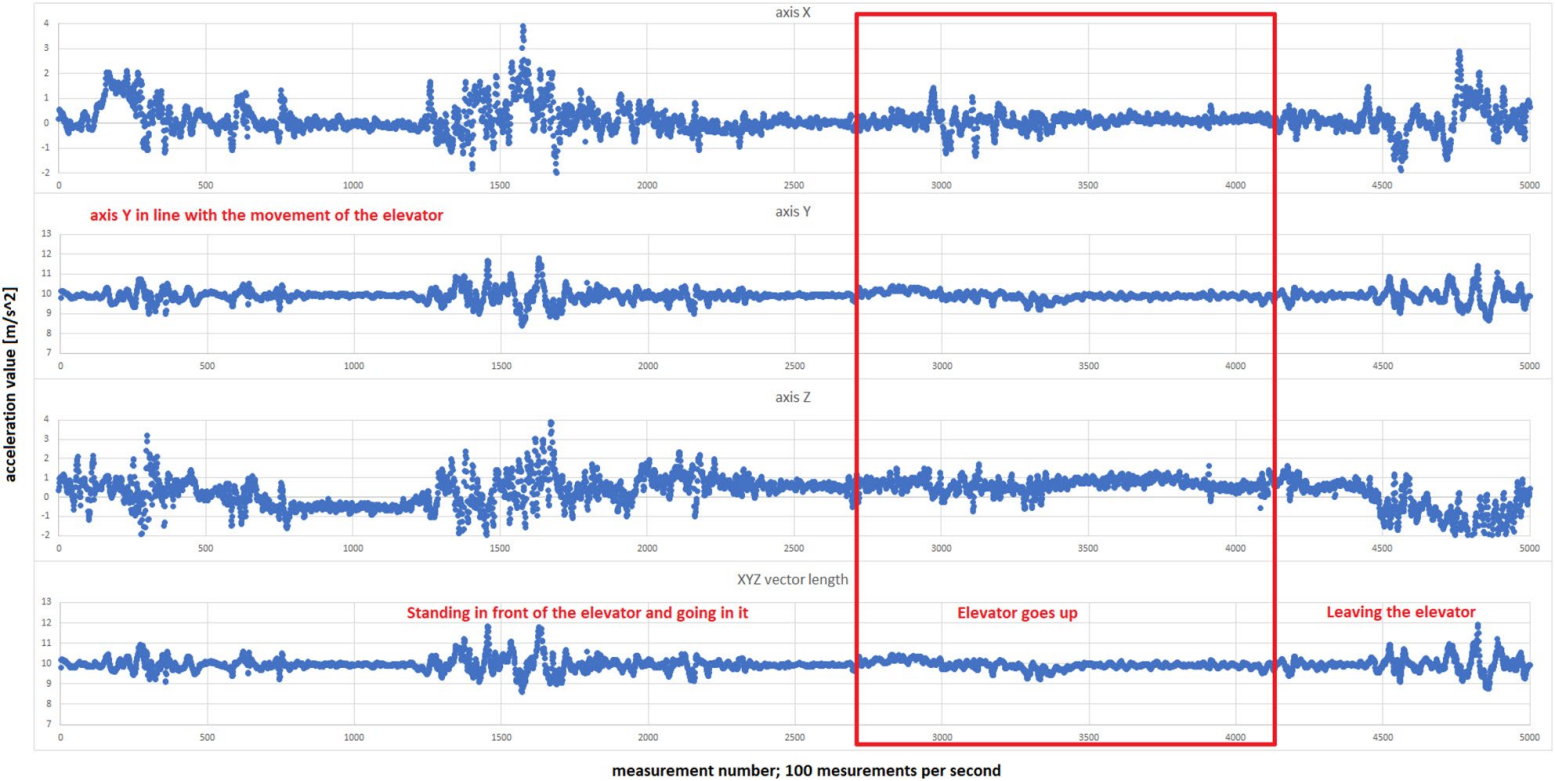
Example of the accelerometer measurements taken at the PAS Institute.

Our framework incorporates building entry detection, elevator ride recognition, and floor number change detection methods. Results indicate high accuracy, with building entry exceeding 93%, elevator recognition achieving 75% sensitivity and 97% specificity, and floor change detection surpassing 95% sensitivity and nearly 98% specificity (which translates to nearly 97% accuracy). Notably, the algorithm is infrastructure-independent, relying on the smartphone’s barometer, and versatile, detecting elevator travel when Wi-Fi AP and LTE are available. Our innovative approach signifies a promising solution for accurate and practical floor-level detection in diverse indoor environments. This framework could be turned into an application that could serve as an advanced employee movement monitor within a multi-building corporate environment. Leveraging our algorithm, the app will accurately track employees’ vertical movements, providing real-time floor-level information. This could aid in optimizing workplace efficiency, enhancing security measures, and facilitating seamless navigation. Future functionalities may include integrating various building infrastructures, enabling personalized notifications, and supporting emergency response scenarios (notably building evacuation), ultimately fostering a dynamic and responsive workplace environment.

The resource requirements of the presented algorithm concept should not be a challenge for today’s smartphones. While current devices can process images in real time and increasingly have hardware support for AI algorithms, our concept in its basic version requires performing fairly simple calculations. It can be assumed that a phone capable of navigating a user (e.g., Google Maps) has adequate hardware resources to read real-time GPS coordinates outside a building, and be able to read and calculate pressure differences using the built-in barometer. Such a device should also easily allow the implementation of auxiliary functions, such as WiFi network detection.

Future works will concentrate on expanding our algorithm, involving additional sensor integration and other algorithm incorporation to enhance precision and functionality even further, including horizontal locating solutions. We can refine indoor positioning by incorporating technologies like inertial sensors, Wi-Fi fingerprinting, or Bluetooth beacons. The algorithms developed in the future can merge data from these sensors, providing comprehensive location awareness. This development promises a more versatile and accurate system, catering to a broader range of applications, from detailed indoor navigation to context-aware workplace optimization.

The introduction to the envisioned further work is the research carried out by the authors on integrating the accelerometer. In its concept, this research aimed to improve the indications of the elevator ride recognition method. The expected effect was that the accelerometer recorded changes in acceleration in the vertical direction while riding the elevator. However, the measurements (see Fig. 13) did not show the expected change in the recorded acceleration. Moreover, the acceleration generated when entering and exiting the elevator was significantly greater. Riding an elevator generates acceleration changes comparable to standing still before entering the elevator (a person does not stand entirely still waiting for the elevator). This makes it impossible to distinguish elevator trips using the accelerometer effectively.

## Figures and Tables

**Figure 14:**
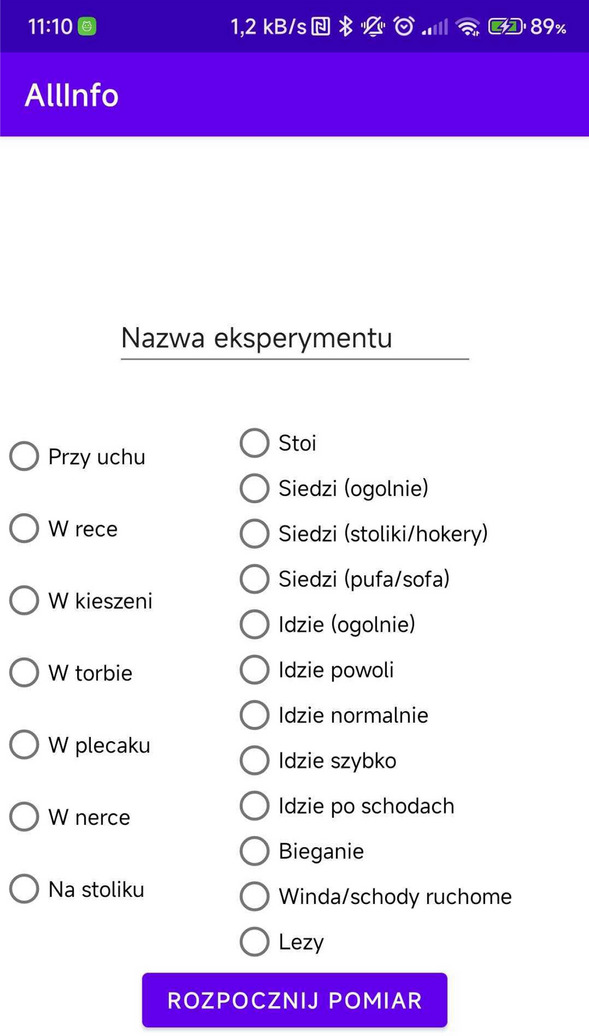
AllInfo application interface.

## Data Availability

The datasets generated and/or analyzed during the current study are available in the record^36^ on the Zenodo.org. The application used to get these data is available on the Github repository: https://github.com/iitis/AllInfoApp.

## Appendix A: Testbed software

The created application integrates various modules to capture a wide range of data, including GPS, LTE, Wi-Fi, and physical sensor readings. This comprehensive approach enables a thorough investigation of GPS signal variations and the correlation of recorded pressure data with building floor levels, which is of significant interest in environmental and urban studies.

### Application interface

The application’s main screen, as shown in Fig. 14, offers a straightforward mechanism for users to input experimental data and select context-specific variables. At the top, a field labeled ‘Nazwa eksperymentu‘ allows the entry of the experiment’s name or other relevant identifiers. Below, the interface provides a set of radio buttons categorized into two groups, enabling users to specify the location of the phone during the experiment (such as ‘Przy uchu‘—by the ear, ‘W rece‘—in hand, and so on) and the current activity of the subject (such as standing, sitting, walking, etc.). The bottom of the screen features a button to start the data collection process, labeled ‘ROZPOCZNIJ POMIAR‘, translating to ‘START MEASUREMENT‘, which, when activated, initiates the sensor data logging. The layout is designed to minimize user input errors and streamline the data collection process.

#### Application architecture

The application’s architecture is modular, comprising several key components and files, each responsible for specific tasks related to data collection and processing. Some most important files are:

- **MainActivity.java** The MainActivity.java serves as the entry point for the application, initiating the user interface and facilitating user interaction. It orchestrates the overall functioning of the app, linking the user interface with underlying data collection services.
- **AllInfoBackground.java** AllInfoBackground.java is designed for background operations, enabling continuous data collection even when the application is not in active use. This feature is crucial for long-term data acquisition scenarios.
- **AllMeasurements.java** This module, AllMeasurements.java, aggregates data from various sensors. It acts as a central hub for collecting GPS coordinates, Wi-Fi and LTE signal strengths, and readings from motion and pressure sensors.
- **GPSsatInfo.java** The GPSsatInfo.java focuses on acquiring detailed GPS data, including satellite information. This is integral for analyzing GPS signal strength and its fluctuations in different environmental conditions.
- **LTECellInfo.java** LTECellInfo.java collects data regarding the LTE network, providing insights into cellular signal quality and variability, which can be correlated with other sensory data for comprehensive analysis.
- **WifiMeasurement.java** In WifiMeasurement.java, Wi-Fi network data is captured, aiding in indoor localization where GPS accuracy diminishes. This module enhances the application’s utility in complex urban environments.
- **FloatXYZ.java** FloatXYZ.java handles three-dimensional sensor data, such as from accelerometers and gyroscopes. This allows for the analysis of device orientation and movement, adding another layer of contextual information to the sensor data.

#### List of sensors and update interval

**Table:**

Sensor	Update frequency (Hz)
Accelerometer	100.0
Gyroscope	100.0
Magnetic Field Sensor	100.0
Light Sensor	2.0
Pressure Sensor	1.0
Screen On	2.0
GPS	1.0
Wi-Fi	1.0
LTE	1.0

In summary, the application provides a robust platform for extensive sensor data collection from smartphones. Its modular architecture and comprehensive data collection capabilities make it an invaluable tool for studying environmental conditions, particularly in urban settings. The integration of GPS, LTE, Wi-Fi, and various physical sensors allows for a multi-faceted approach to data analysis, essential for understanding the complex interplay of environmental factors in modern urban landscapes.
